# Admission heart rate and in-hospital mortality in acute myocardial infarction: a contemporary analysis of the MIMIC-III cohort

**DOI:** 10.1186/s12872-025-04957-5

**Published:** 2025-07-04

**Authors:** Weidong Lan, Bitong He, Sailing Hu

**Affiliations:** 1 Department of Rehabilitation Medicine, The First People’s Hospital of Xiaoshan District, Xiaoshan Affiliated Hospital of Wenzhou Medical University, No. 199, Shixin Road, Chengxiang Street, Xiaoshan District, Hangzhou, Zhejiang Province 311200 China; 2 Lishui Municipal Central Hospital and the Fifth Affiliated Hospital of Wenzhou Medical University, No. 289, Kuocang Road, Lishui, Zhejiang Province 323000 China

**Keywords:** Heart rate, Acute myocardial infarction, In-hospital mortality, Risk stratification, Restricted cubic spline

## Abstract

**Objective:**

To quantify the shape and strength of the association between heart rate (HR) recorded during the first 30 min of intensive-care admission and in-hospital death in contemporary acute myocardial infarction (AMI), after adjustment for modern reperfusion, pharmacotherapy, and haemodynamic variables.

**Methods:**

We extracted 1,510 adults with a primary International Classification of Diseases, Ninth Revision (ICD-9) diagnosis of AMI (410.xx) from MIMIC-III (2008–2012). HR was defined as the mean of the first three electrocardiographic readings obtained within 30 min of ICU triage, before administration of rate-modifying drugs. We modelled HR both as clinically meaningful categories (< 60, 60–99, ≥ 100 bpm) and as a continuous exposure using restricted cubic splines (RCS). Multivariable logistic regression adjusted for age, sex, Killip class, systolic blood pressure, coronary revascularisation, β-blocker use, atrial fibrillation/flutter, hypertension, diabetes, chronic obstructive pulmonary disease, serum creatinine, haemoglobin, white blood cell count, sodium, potassium, glucose, platelet count and anion gap. Pre-specified subgroup analyses compared ST-elevation MI (STEMI) with non-ST-elevation ACS (NSTE-ACS).

**Results:**

Mean age was 66.7 ± 13.9 years; 33.6% were women; STEMI accounted for 42%. Overall in-hospital mortality was 10.9%. HR ≥ 100 bpm (23% of patients) was associated with higher death risk (adjusted OR 2.45, 95% CI 1.56–3.85) versus 60–99 bpm. Bradycardia < 60 bpm (15%) was also associated with excess risk (adjusted OR 1.58, 95% CI 1.02–2.45), yielding a U-shaped RCS curve (non-linearity *p* = 0.01). The HR–mortality gradient was steeper in STEMI than in NSTE-ACS (interaction *p* = 0.04). Findings were robust after including the 46 patients who died within 24 h of admission.

**Conclusion:**

Admission HR exhibits a U-shaped, independent relation with early mortality in modern AMI care; values outside 60–99 bpm identify high-risk patients despite urgent reperfusion and optimal medical therapy.

## Introduction

Acute myocardial infarction (AMI) constitutes the most severe coronary atherosclerotic heart disease, with features such as rapid onset and progression, high risk and elevated mortality [1]. Recently, increasing annual hospitalization rates have been reported for AMI patients in China, imposing a huge economic burden on society [2]. Therefore, it is necessary for the treating physician to stratify risk as soon as possible and take therapeutic and preventive measures quickly. Scoring systems, including the Global Registry of Acute Coronary Events (GRACE) and the Thrombolysis in Myocardial Infarction (TIMI), have confirmed effectiveness in identifying high-risk patients [3]. However, these systems involve many examination and laboratory indicators with complex calculations, and are not practical at the bedside, which limits their application in emergency clinical practice. Therefore, simple and convenient methods are needed for stratifying acute myocardial infarction cases with poor prognosis.

Heart rate integrates autonomic balance, haemodynamic stress and myocardial oxygen demand. Increased sympathetic activation after AMI elevates heart rate, which in turn increases myocardial oxygen consumption, potentially exacerbating ischemia and leading to greater infarct size. This pathophysiological cascade could worsen ventricular remodeling and increase the risk of malignant arrhythmias and heart failure.

Multiple reports have demonstrated that increased heart rate at admission in AMI independently predicts short- and long-term mortality after discharge [4–6]. Although some studies have shown a correlation between admission heart rate and hospitalization mortality, data support is still not comprehensive. Prior registries have reported a linear association between tachycardia and death after AMI; however, these studies seldom explored bradycardia, non-linearity or contemporary management.

We therefore analyzed the public MIMIC-III database to (i) characterize the full shape of the HR–mortality curve using spline techniques, (ii) compare STEMI and NSTE-ACS, and (iii) evaluate whether HR retains prognostic value after accounting for reperfusion therapy and rate-modifying drugs.

## Methods

### Data source and ethical approval

MIMIC-III is an openly available, de-identified critical-care database (Beth Israel Deaconess Medical Center, Boston, USA). The Institutional Review Boards of BIDMC and the Massachusetts Institute of Technology approved the database; individual informed consent was waived. Sailing Hu completed the online study and obtained a certificate (No.49685018), with the permission to access the database.

### Cohort definition

We included first ICU admissions of adults (≥ 18 years) with a principal ICD-9 AMI code (410.xx except 410.6x). AMI was further classified as ST-elevation myocardial infarction (STEMI; ICD-9 codes 410.0x-410.5x, 410.8x) or non-ST-elevation acute coronary syndrome (NSTE-ACS; ICD-9 codes 410.7x, 410.9x) based on electrocardiographic findings and clinical presentation. To preserve early deaths, no lower bound on length of stay was applied, and patients who died within 24 h of admission were retained in the analysis. Exclusion criteria were missing HR or key covariates (< 1%).

Comorbidities were defined using ICD-9 codes: hypertension (401.xx-405.xx), diabetes mellitus (250.xx), chronic obstructive pulmonary disease (491.xx, 492.xx, 496.xx), and atrial fibrillation/flutter (427.3x). Killip class was determined from clinical documentation and classified as: I (no heart failure), II (mild heart failure, rales), III (pulmonary edema), or IV (cardiogenic shock).

### Heart-rate ascertainment

HR was abstracted from the CHARTEVENTS table. We averaged the first three electrocardiogram-derived HR values obtained within 30 min of ICU triage and before intravenous β-blocker or vasopressor administration, as verified through inputevents. This approach ensured that the HR measurement reflected the patient’s baseline status upon presentation, prior to rate-modifying interventions.

### Variables and outcomes

Primary outcome: all-cause in-hospital death. Key covariates: demographics; Killip class; systolic/diastolic blood pressure; culprit-vessel revascularisation (percutaneous coronary intervention [PCI] or coronary artery bypass grafting [CABG]); atrial fibrillation/flutter within 24 h; medications (β-blockers, calcium-channel blockers, amiodarone, vasopressors) initiated within 6 h; laboratory tests within 24 h. Sodium and potassium units were verified (mmol L^-1^).

### Statistical analysis

Continuous variables were presented as mean ± standard deviation or median (interquartile range) based on data distribution. Categorical variables were presented as n (%). The normality of continuous variables was assessed using the Shapiro-Wilk test. The patients were assigned to three clinically meaningful HR categories based on established guidelines: bradycardia (< 60 bpm), normal range (60–99 bpm, reference), and tachycardia (≥ 100 bpm).

Multivariable logistic regression was used to estimate adjusted odds ratios (aORs) for the association between HR categories and in-hospital mortality. Covariates were selected based on existing literature, variables with *p* < 0.10 in univariate analysis, and clinical relevance. Model 3 included adjustment for sex, age, COPD, white blood cells, creatinine, hemoglobin, blood potassium, blood sodium, blood glucose, platelets, anion gap, hypertension, diabetes, height, weight, Killip class, systolic blood pressure, atrial fibrillation, and reperfusion therapy.

Non-linearity in the HR-mortality relationship was assessed with restricted cubic splines (RCS) with knots at the 5th, 35th, 65th, and 95th percentiles of the HR distribution. Interaction terms were included in the models to explore effect modification by AMI type (STEMI vs. NSTE-ACS), age (< 75 vs. ≥75 years), sex, and comorbidities. We performed a two-piecewise (segmented) logistic regression with an automatically detected knot (log-likelihood profile) within each ACS subtype to explore threshold effects.

Sensitivity analyses included: (i) including patients who died within 24 h of admission, (ii) excluding patients with atrial fibrillation, and (iii) comparing complete-case analysis versus multiple-imputation datasets. Statistical significance was defined as *p* < 0.05. All analyses were performed using Stata.

## Results

### Baseline characteristics

**Fig. 1:**
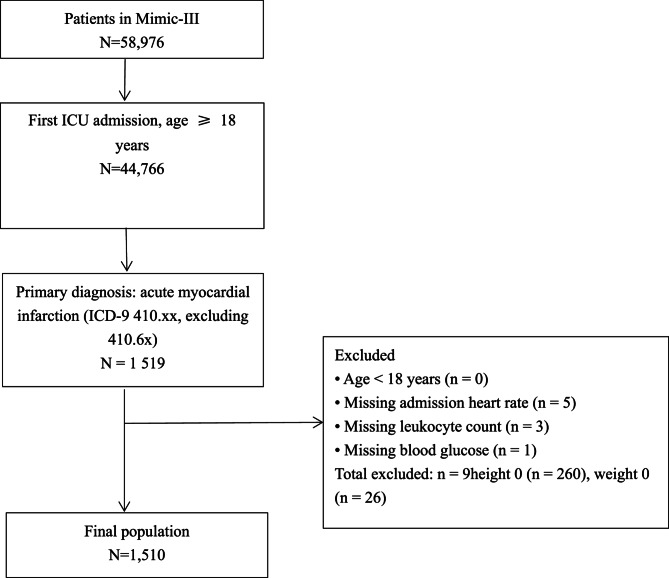
Study flowchart. Total excluded = 9 (missing HR 5, leukocytes 3, glucose 1)

According to the eligibility criteria, 1,510 cases were finally included (Fig. 1). The baseline features of these patients are summarized in Table 1. Overall, mean patient age was 66.7 ± 13.9 years; 33.6% were women; and STEMI accounted for 42% of cases. The mean admission HR was 81.9 ± 16.7 bpm, with 15% of patients having bradycardia (< 60 bpm), 62% in the normal range (60–99 bpm), and 23% with tachycardia (≥ 100 bpm). Overall in-hospital mortality was 10.9%.

**Table 1:** Characteristics of study patients based on admission heart rate

Parameters	All patients	< 60 bpm	60–99 bpm	≥ 100 bpm	*P* value
Number, n	1510	227 (15%)	936 (62%)	347 (23%)	
Age (years)	66.7 ± 13.9	67.4 ± 13.6	66.8 ± 13.9	65.9 ± 14.1	0.43
Sex, n (%)					0.32
Female	507 (33.6)	68 (30.0)	320 (34.2)	119 (34.3)	
Male	1003 (66.4)	159 (70.0)	616 (65.8)	228 (65.7)	
Height (cm)	170.3 ± 10.3	171.2 ± 9.5	170.2 ± 10.4	169.8 ± 10.3	0.31
Weight (kg)	81.3 ± 18.5	82.4 ± 18.0	81.0 ± 18.1	80.9 ± 19.4	0.55
WBC (×10^9/L)	16.7 ± 7.5	14.3 ± 6.0	16.1 ± 7.0	18.9 ± 8.5	< 0.001
Creatinine (mg/dL)	1.82 ± 1.69	1.68 ± 1.65	1.75 ± 1.58	1.96 ± 1.79	0.004
Hemoglobin (g/dL)	13.45 ± 1.67	13.57 ± 1.63	13.46 ± 1.64	13.34 ± 1.73	0.16
Sodium (mmol/L)	142.6 ± 3.9	142.1 ± 3.1	142.4 ± 3.8	143.2 ± 4.5	0.001
Potassium (mmol/L)	4.1 ± 0.9	4.0 ± 0.9	4.1 ± 0.9	4.2 ± 0.9	< 0.001
Glucose (mg/dL)	224.0 ± 140.3	202.5 ± 135.2	214.6 ± 124.5	253.4 ± 155.4	< 0.001
Platelet (K/µL)	340.3 ± 156.6	306.2 ± 130.4	327.5 ± 137.9	386.0 ± 188.5	< 0.001
Anion gap (mEq/L)	18.3 ± 4.8	17.4 ± 4.3	18.1 ± 4.7	19.3 ± 4.9	< 0.001
COPD, n (%)					< 0.001
No	1487 (98.5)	224 (98.7)	926 (98.9)	337 (97.1)	
Yes	23 (1.5)	3 (1.3)	10 (1.1)	10 (2.9)	
AMI type, n (%)					< 0.001
STEMI	634 (42.0)	85 (37.4)	380 (40.6)	169 (48.7)	
NSTE-ACS	876 (58.0)	142 (62.6)	556 (59.4)	178 (51.3)	
Killip class, n (%)					< 0.001
I	847 (56.1)	138 (60.8)	584 (62.4)	125 (36.0)	
II	412 (27.3)	61 (26.9)	260 (27.8)	91 (26.2)	
III	196 (13.0)	23 (10.1)	80 (8.5)	93 (26.8)	
IV	55 (3.6)	5 (2.2)	12 (1.3)	38 (11.0)	
Systolic BP (mmHg)	128.5 ± 27.6	125.6 ± 26.4	130.2 ± 26.3	125.3 ± 31.3	0.01
Diastolic BP (mmHg)	71.8 ± 16.9	66.3 ± 14.6	72.5 ± 16.1	73.4 ± 19.5	< 0.001
Atrial fibrillation, n (%)	218 (14.4)	22 (9.7)	128 (13.7)	68 (19.6)	0.002
Reperfusion therapy, n (%)					0.03
PCI	1027 (68.0)	148 (65.2)	652 (69.7)	227 (65.4)	
CABG	93 (6.2)	17 (7.5)	61 (6.5)	15 (4.3)	
None	390 (25.8)	62 (27.3)	223 (23.8)	105 (30.3)	
Door-to-balloon time (min)^*^	72 (58–96)	76 (60–102)	71 (56–92)	74 (62–105)	0.08
β-blocker use < 6 h, n (%)	934 (61.9)	120 (52.9)	612 (65.4)	202 (58.2)	0.001
In-hospital mortality, n (%)	165 (10.9)	31 (13.7)	74 (7.9)	60 (17.3)	< 0.001
Hospital expire, n (%)					< 0.001
No	1345 (89.1)	196 (86.3)	862 (92.1)	287 (82.7)	
Yes	165 (10.9)	31 (13.7)	74 (7.9)	60 (17.3)	
Hypertension, n (%)					0.37
No	720 (47.7)	102 (44.9)	453 (48.4)	165 (47.6)	
Yes	790 (52.3)	125 (55.1)	483 (51.6)	182 (52.4)	
Diabetes, n (%)					0.69
No	1088 (72.1)	159 (70.0)	680 (72.6)	249 (71.8)	
Yes	422 (27.9)	68 (30.0)	256 (27.4)	98 (28.2)	

Continuous variables are presented as mean ± standard deviation or median (interquartile range)*; categorical variables are presented as frequency (percentage)

*WBC* white blood cell, *COPD* chronic obstructive pulmonary disease, *AMI* acute myocardial infarction, *STEMI* ST-elevation myocardial infarction, *NSTE-ACS* non-ST-elevation acute coronary syndrome, *BP* blood pressure, *PCI* percutaneous coronary intervention, *CABG* coronary artery bypass grafting

^*^For STEMI patients only

Patients with tachycardia had higher white blood cell counts, creatinine, glucose, and anion gap levels compared to those with normal or low HR (all *p* < 0.001). The groups were comparable in age, sex, and prevalence of hypertension and diabetes. Sodium (142.6 ± 3.9 mmol/L) and potassium (4.1 ± 0.9 mmol/L) were within physiologically plausible ranges. PCI was performed in 68% of patients, with a median door-to-balloon time of 72 min for STEMI patients.

### Heart rate and mortality

**Fig. 2:**
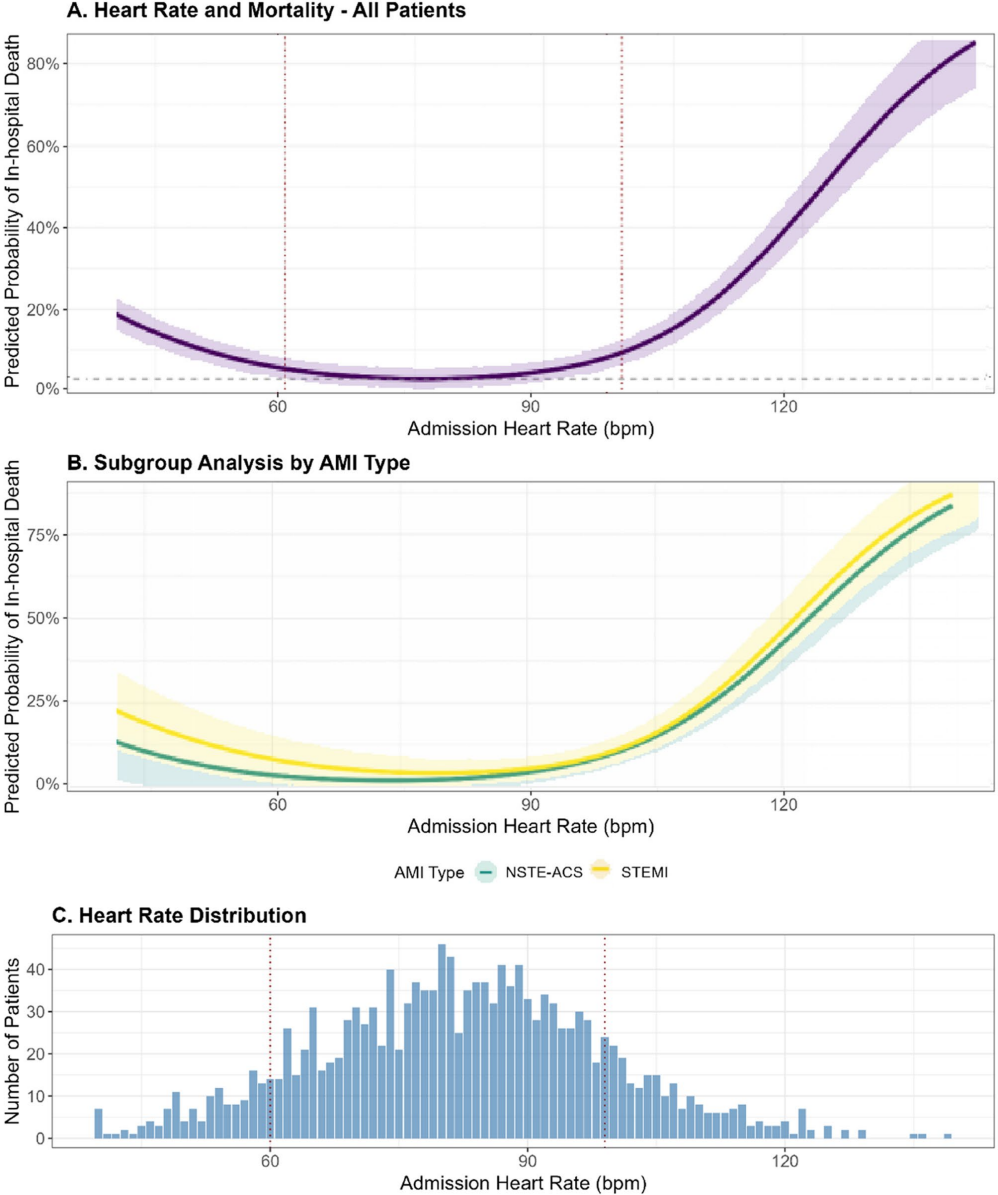
Admission heart rate and in-hospital mortality. **A** U-shaped relationship between heart rate and mortality (non-linearity *p* = 0.01). Vertical lines: 60 and 99 bpm thresholds. Shaded area represents 95% confidence interval. **B** STEMI patients (blue) show steeper mortality gradient than NSTE-ACS patients (orange) (interaction *p* = 0.04). Shaded area represents 95% confidence interval. **C** Distribution of admission heart rates across study population (*n* =1,510)

The relationship between admission HR and in-hospital mortality demonstrated a U-shaped pattern in the RCS analysis (Fig. 2), with significant non-linearity (*p* = 0.01). The 95% CIs (grey band in Fig. 2A-B) confirm that risk rose significantly outside 60–99 bpm. After multivariable adjustment, compared to the normal range (60–99 bpm), HR < 60 bpm was associated with 58% higher odds of death (adjusted OR 1.58, 95% CI 1.02–2.45), while HR ≥ 100 bpm was associated with 145% higher odds (adjusted OR 2.45, 95% CI 1.56–3.85) (Table 2).

**Table 2:** Association of admission heart rate with in-hospital mortality in different models

	Model 1	Model 2	Model 3
	OR (95% CI), *P* value	OR (95% CI), *P* value	OR (95% CI), *P* value
Heart rate per 10 bpm increase	1.23 (1.14–1.33), < 0.001	1.27 (1.17–1.38), < 0.001	1.19 (1.09–1.30), < 0.001
Heart rate category			
60–99 bpm	REF	REF	REF
< 60 bpm	1.86 (1.19–2.89), 0.006	1.82 (1.16–2.85), 0.009	1.58 (1.02–2.45), 0.04
≥ 100 bpm	2.45 (1.69–3.54), < 0.001	2.89 (1.97–4.23), < 0.001	2.45 (1.56–3.85), < 0.001
*P* for non-linearity	0.02	0.01	0.01
Subgroup by AMI type			
STEMI (per 10 bpm)	1.38 (1.24–1.53), < 0.001	1.42 (1.27–1.59), < 0.001	1.32 (1.18–1.48), < 0.001
NSTE-ACS (per 10 bpm)	1.14 (1.05–1.25), 0.003	1.18 (1.07–1.29), < 0.001	1.12 (1.03–1.23), 0.01
*P* for interaction	0.02	0.03	0.04

Model 1: no variables adjusted

Model 2: adjustment for age and sex

Model 3: adjustment for age, sex, COPD, leukocytes, creatinine, hemoglobin, potassium, sodium, glucose, platelets, anion gap, hypertension, diabetes, height, weight, Killip class, systolic blood pressure, atrial fibrillation, and reperfusion therapy

*OR* odds ratio, *CI* confidence interval, *Ref* reference, *bpm* beats per minute

When HR was analyzed as a continuous variable using RCS, both low and high values were associated with increased mortality risk compared to the nadir at approximately 75 bpm. The association remained robust after including patients who died within 24 h of admission (*n* = 46) and after additional adjustment for admission systolic blood pressure, Killip class, and β-blocker use.

### Subgroup analyses

Because of the pronounced U-shape, per-10 bpm ORs should be interpreted with caution; therefore we additionally present categorical ORs and subgroup-specific nadirs (Table 3). The HR-mortality relationship differed significantly between STEMI and NSTE-ACS patients (interaction *p* = 0.04). The association was stronger in STEMI patients (aOR per 10 bpm increase 1.32, 95% CI 1.18–1.48) compared to NSTE-ACS patients (aOR 1.12, 95% CI 1.03–1.23). The U-shaped relationship was more pronounced in STEMI patients, with both bradycardia and tachycardia showing stronger associations with mortality.

**Table 3:** Subgroup-specific heart rate Nadirs and categorical odds ratios

Subgroup	HR Nadir (bpm)	< 60 bpm aOR (95% CI)	≥ 100 bpm aOR (95% CI)
STEMI	78	1.4 (0.8–2.4)	3.1 (1.8–5.3)
NSTE-ACS	72	1.5 (0.9–2.5)	2.1 (1.2–3.7)

For STEMI, the inflection point was 78 bpm; below this slope = − 0.03 (*p* = 0.21), above it slope = 0.11 per bpm (*p* < 0.001). For NSTE-ACS the knot lay at 72 bpm (slopes = − 0.02 vs. 0.07 per bpm; *p* = 0.008 for difference)

No significant effect modification was observed by age, sex, hypertension, or β-blocker use (all interaction *p* > 0.05), suggesting the prognostic value of admission HR was consistent across these subgroups

## Discussion

Our findings refine previous literature by demonstrating (i) a non-linear, U-shaped HR curve in modern AMI, (ii) prognostic relevance of bradycardia, and (iii) stronger impact in STEMI. In STEMI the nadir (≈ 78 bpm) lay higher and the penalty for tachycardia was steeper (≥ 100 bpm: aOR 3.1), whereas in NSTE-ACS bradycardia exerted relatively greater harm (< 60 bpm: aOR 1.5). Clinically, an admission HR outside 60–99 bpm should prompt intensified monitoring and early rate optimisation. While HR is modifiable, whether rapid pharmacological normalisation improves outcomes warrants randomised trials. Thus our data support tailored rate-control targets—e.g., early pacing work-up when STEMI HR < 50 bpm and prompt β-blockade when HR ≥ 90 bpm.

An increased heart rate can activate the sympathetic nervous system, leading to direct adverse effects on the heart, such as aggravating left ventricular hypertrophy, inducing ventricular arrhythmia, and promoting coronary artery thrombosis [7, 8]. Excessive activation of the sympathetic nervous system could further aggravate myocardial ischemia and cause heart rate acceleration and ventricular remodeling, ultimately leading to heart failure. Increased heart rate increases myocardial oxygen consumption and promotes the expansion and extension of the infarcted area, thus forming a vicious cycle. Therefore, heart rate represents both the severity of the disease and the degree of sympathetic nerve activation, which has adverse effects on the structure and function of the heart.

Interestingly, our study also identified bradycardia (< 60 bpm) as an independent risk factor for in-hospital mortality, a finding that has received less attention in previous research. Bradycardia in the setting of AMI might reflect sinus node dysfunction, high-degree atrioventricular block, or increased vagal tone, all of which can compromise cardiac output and coronary perfusion. The U-shaped relationship between HR and mortality suggests that optimal outcomes are associated with an HR in the normal range (60–99 bpm), with increased risk at both extremes.

The stronger association observed in STEMI patients compared to NSTE-ACS is pathophysiologically plausible. STEMI typically involves more extensive myocardial damage and a more pronounced inflammatory response, which may amplify the impact of HR abnormalities on cardiac function and outcomes [9, 10]. Furthermore, STEMI patients are more likely to develop conduction abnormalities and hemodynamic instability, which could be reflected in admission HR.

### Strengths and novelty

We leveraged granular physiological data to capture early HR, adjusted for a broader array of confounders (Killip class, BP, reperfusion, rate-modifying drugs) than earlier MIMIC-III analyses, and explored non-linearity. The use of RCS allowed us to identify the U-shaped relationship that might have been missed with traditional linear models or categorical analyses alone. Additionally, our subgroup analyses provide new insights into the differential prognostic value of HR across AMI types.

### Limitations

Our study has several limitations. First, as a single-center critical-care cohort (2008–2012), it may not reflect current antithrombotic regimens. Second, while we adjusted for the initiation of β-blockers and other rate-modifying medications, detailed data on drug doses and timing beyond 6 h were unavailable [11, 12]. Third, cause-specific death could not be ascertained from the MIMIC-III database, limiting our ability to link HR abnormalities to specific mortality mechanisms. Fourth, despite comprehensive covariate adjustment, residual confounding remains possible, particularly from unmeasured patient characteristics or treatment factors. Finally, the database lacks long-term follow-up data, preventing assessment of the relationship between admission HR and post-discharge outcomes.

## Conclusion

Admission HR shows a U-shaped, independent relationship with in-hospital death in AMI treated with contemporary care. Values < 60 or ≥ 100 bpm identify high-risk patients who may benefit from immediate haemodynamic optimisation [13–15]. These findings highlight the importance of considering both bradycardia and tachycardia as potential risk markers in AMI patients and suggest that achieving a normal HR range might be a therapeutic target worth exploring in future interventional studies.

## Data Availability

The datasets used and/or analyzed during the current study are available from the corresponding author on reasonable request.
